# Vector Competence of Italian Populations of *Culicoides* for Some Bluetongue Virus Strains Responsible for Recent Northern African and European Outbreaks

**DOI:** 10.3390/v11100941

**Published:** 2019-10-12

**Authors:** Valentina Federici, Maria Goffredo, Giuseppe Mancini, Michela Quaglia, Adriana Santilli, Francesca Di Nicola, Matteo De Ascentis, Pierangela Cabras, Carmela Volpicelli, Claudio De Liberato, Giuseppe Satta, Giovanni Federico, Alessandra Leone, Maura Pisciella, Ottavio Portanti, Federica Pizzurro, Liana Teodori, Giovanni Savini

**Affiliations:** 1 Istituto Zooprofilattico Sperimentale dell’Abruzzo e del Molise ‘G. Caporale’, Via Campo Boario, 64100 Teramo, Italy; m.goffredo@izs.it (M.G.); g.mancini@izs.it (G.M.); m.quaglia@izs.it (M.Q.); a.santilli@izs.it (A.S.); f.dinicola@izs.it (F.D.N.); m.deascentis@izs.it (M.D.A.); a.leone@izs.it (A.L.); m.pisciella@izs.it (M.P.); o.portanti@izs.it (O.P.); f.pizzurro@izs.it (F.P.); l.teodori@izs.it (L.T.); g.savini@izs.it (G.S.); 2 Istituto Zooprofilattico Sperimentale della Sardegna, Via Duca degli Abruzzi 8, 07100 Sassari, Italy; pierangela.cabras@izs-sardegna.it (P.C.); giuseppe.satta@izs-sardegna.it (G.S.); 3 Azienda Sanitaria Provinciale Crotone, Via M. Nicoletta, 88900 Crotone (KR), Italy; veterinari.areaa@asp.crotone.it; 4 Istituto Zooprofilattico Sperimentale del Lazio e della Toscana, Via Appia Nuova 1411, 00178 Rome, Italy; claudio.deliberato@izslt.it; 5 Istituto Zooprofilattico Sperimentale del Mezzogiorno, Via Figurella, 89135 Catona (RC), Italy; giovanni.federico@izsmportici.it

**Keywords:** *Culicoides imicola*, *Culicoides obsoletus*, *Culicoides scoticus*, bluetongue virus, oral infection, vector competence, recovery rate, Italy

## Abstract

The distribution of Bluetongue virus (BTV) in Europe can be represented by two distinct and interconnected epidemiological systems (episystems), each characterized by different ecological characteristics and vector species. This study investigated the vector competence of Italian populations of *Culicoides imicola* and *Culicoides obsoletus/scoticus* to some representative BTV strains after artificial oral infection. The BTV strains were selected according to their ability to spread to one or both episystems and included BTV-4 ITA, responsible of the recent Italian and French BTV-4 outbreaks; the BTV-2 strain which caused the first BTV incursion in Italy, Corsica, and Balearic Islands; BTV-4 MOR, responsible for the epidemic in Morocco; and BTV-8, the strain which spread through Europe between 2006 and 2008. Blood-soaked cotton pledgets and Hemotek membrane feeder using Parafilm^®^ membrane were used to artificially feed midges. For each population/strain, recovery rates (positive/tested heads) were evaluated using serogroup- and serotype-specific RT-PCR. The trial demonstrated that, except for the Abruzzo population of *C. obsoletus/C. scoticus*, which was refractory to BTV-4 MOR infection, all the investigated *Culicoides* populations are susceptible to the selected BTV strains and that, if prompt vaccination programs and restriction measures had not been implemented, BTV-2 and BTV-4 MOR could have spread all over Europe.

## 1. Introduction

*Culicoides* biting midges (Diptera: Ceratopogonidae) are biological vectors of several important arboviruses such as those causing Bluetongue (BT), African horse sickness, and Epizootic hemorrhagic disease. Because of the direct effect of the disease on animals, but especially because of the ensuing ban on international trade of ruminants and their products between BTV-infected and non-infected areas, outbreaks of Bluetongue virus (BTV) continue to have significant economic impacts in Europe and worldwide. In relation to the spread of BTV strains, two distinct and interconnected epidemiological systems (episystems), with different ecological characteristics and vector species, have been identified in Europe. The concept of episystem includes the set of biological, environmental, and epidemiological elements of a disease in defined geographic and temporal scales [1]. For BT, it is known that distinct strains of BTV (virus topotypes) vectored by different species of *Culicoides* occur in specific regions of the world. The topotypes of BTV and the vector species that occur within each episystem are relatively stable, despite extensive and ongoing trade and movement of ruminants between individual episystems.

One episystem is distributed in Southern Europe (Mediterranean Basin), where BTV is primarily spread by *Culicoides imicola*, and one in Southern, Central, and Northern Europe, in which species of the Obsoletus complex act as main vectors [2,3,4]. In the recent European incursions, some BTV strains were able to successfully spread and adapt in both episystems, while some other strains remained confined to one episystem only. It has been hypothesized that this capacity to spread and adapt to different episystems is somehow related to the vector competence of the *Culicoides* species present. It has been observed that the BTV spreading in the US western states was strictly associated to the different distribution of the two more common indigenous species, *Culicoides sonorensis* and *Culicoides variipennis*, and to their different vector competence [5]. Due to its particular geographic position, Italy comprises both episystems. *Culicoides imicola* and species of the Obsoletus complex are geographically complementary and together cover the whole Italian peninsula [6].

The Obsoletus complex is a monophyletic group. In Italy, it is composed almost exclusively by two species, *Culicoides obsoletus* and *Culicoides scoticus*, although a third species, *Culicoides montanus*, is rarely recorded. This study aimed to investigate the vector competence of different Italian populations of *Culicoides imicola* and *C. obsoletus/scoticus* to some BTV strains responsible for recent incursions in the Mediterranean basin and Europe after artificial oral feeding. Four BTV strains (BTV-4 ITA 2014, BTV-2 2001, BTV-4 MOR, BTV-8 2006) were selected based on their ability to spread to one or two episystems.

In Italy, BTV appeared for the first time in Sardinia in 2000. This incursion, due to BTV-2, caused severe clinical signs and death in the affected sheep. BTV-2 then rapidly spread to Sicily and to other regions of South-Central Italy (Basilicata, Lazio, Campania, and Tuscany) [7]. Even if the most implicated vector of the Sardinian BTV-2 outbreaks was *C. imicola,* other *Culicoides* species, including *C. obsoletus* and *C. scoticus,* contributed to the BTV spread in Italy [8]. BTV-2 was in fact isolated from species of the Obsoletus complex caught in Campania and Apulia regions [9]. BTV-2 has never been reported in Central-Northern Europe. Since this first occurrence, several other BTV incursions were reported in the Italian territory, involving serotypes 1, 2, 4, 9, 16, 8, and 3 [10,11]. The second BTV strain selected for this study was the BTV-8 responsible for the most severe BT epidemic ever reported in Europe. After emerging in Central Europe in 2006, it further spread to Northern and Southern European countries. It appeared in Italy in 2008, invading Northern Italy and the Sardinia region in 2009. The third selected BTV strain was the BTV-4, originating from the Balkan peninsula, which has been responsible for the recent Italian (2014–2019) and French (2018–2019) epidemics. This strain has been detected in pools of the Obsoletus complex (Apulia) [3] and in pools of *C. imicola* in Sardinia in 2017 (unpublished data). Finally, the fourth selected strain was a BTV-4 reassortant strain, containing sequences from previous BTV-1 (MOR2006/06) and BTV-4 (MOR 2004/02) strains that spread in Morocco and neighboring regions. It circulated in Morocco in 2009 [12] but was never reported in Italy or in Central-Northern Europe. With a positive correlation between disease and its abundance/distribution, *C. imicola* was suggested to be the main vector species [13].

The *Culicoides* populations included in this study were selected based on episystems and presence/abundance of indigenous species: *C. imicola* of the Sardinia and Calabria regions was selected as representative of the Southern Europe episystem and *C. obsoletus/scoticus* of the Abruzzo and Lazio regions as representative of the other episystem.

In this study, two artificial blood feeding methods were used: the Hemotek membrane system and cotton wool pledgets. Because of their reluctance to feed under laboratory conditions, few artificial oral feeding and BTV vector competence studies have been carried out on species of the Obsoletus complex [14,15,16,17,18]. By contrast, as it is relatively easy to feed *C. imicola* through a membrane and via cotton wool pledgets [19], the susceptibility of *C. imicola* to a variety of BTV strains has been frequently determined in the lab [20,21,22,23,24]. In this study, the feeding rate and the vector competence for both taxa were investigated.

## 2. Materials and Methods 

### 2.1. Collection Sites

**Figure 1:**
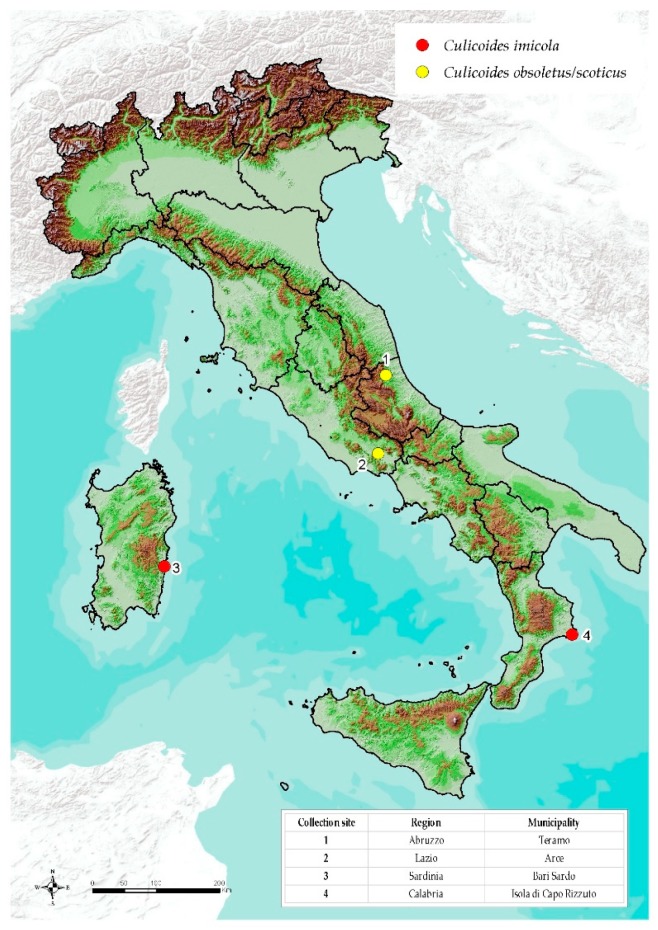
Location of the Italian population of *Culicoides obsoletus/scoticus* (Abruzzo and Lazio regions) and *Culicoides imicola* (Sardinia and Calabria regions).

Collections of *Culicoides* were made in four Italian regions (Abruzzo, Lazio, Sardinia, and Calabria) at the selected farms (Figure 1). The suitable collection sites were selected based on *Culicoides* abundance results derived from the entomological National surveillance plan implemented in Italy since 2000. The insect collections were performed by the team of the Istituto Zooprofilattico Sperimentale dell’Abruzzo e del Molise “G. Caporale” (IZSAM), in collaboration with the network of the Italian Istituti Zooprofilattici Sperimentali (IIZZSS) and the local Veterinary Services.

### 2.2. Field Activities

On each farm, a variable number (4–7) of UV blacklight suction traps operated all night long from about one hour before dusk on two or three consecutive days. Field *Culicoides* were collected early in the morning by placing the collection beakers in boxes and allowing trapped midges to fly in card glasses through funnels.

Overall, 31 and 30 adult collection nights were performed, from 7 June 2016 to 19 October 2016 and from 20 June 2017 to 18 October 2017, respectively.

Collected *Culicoides* were then transferred in 64 mm card boxes and fed with sucrose solution 10%. After two days of acclimatization at 25 °C and 80% > HR > 40%, they were starved for one day before the oral infection. 

### 2.3. Laboratory Activities

#### 2.3.1. Virus Isolates

For the experimental infection, four BTV strains were selected:

- BTV-2, strain 2000TE8341: It was isolated from the spleen of a Sardinian sheep that died because of BTV in 2000. The strain was passed once onto the Kc cell line and three times onto VERO cells (from African green monkey kidney) before use.
- BTV-4 ITA, strain 2014TE31172: It was isolated from the blood of a sheep infected with BTV in the Apulia region in 2014. The strain was passed once onto the Kc cell line and three times onto VERO cells before use.
- BTV-4 MOR, strain MOR2009-07: Kindly provided by the Pirbright Institute (Lara Harrup), it was isolated from a blood sample of a BTV-infected sheep in Morocco in 2009. This strain was passed three times onto the Kc cell line and twice onto VERO cells before use.
- BTV-8, strain UKG2007-82: Kindly provided by the Pirbright Institute (Lara Harrup), it was isolated from a blood sample of a bovine infected in the UK in 2007. The strain was passed three times onto the Kc cell line and twice onto VERO cells before use.

#### 2.3.2. Culicoides Oral Infection 

All experimental infection phases were performed at IZSAM in a dedicated room within the BSL3 facilities. To obtain the blood meals for infecting *Culicoides*, BTV suspensions were diluted (1:3, 1:4 or 1:5) in defibrinated cattle or sheep blood. *Culicoides* were fed for 45 min using either the Hemotek blood feeding system with a Parafilm^®^ membrane or the cotton pledgets in accordance with the method described by Venter and colleagues [19]. The Parafilm Hemotek system has already been successfully used for feeding field-collected *Culicoides* and vector competence studies, obtaining feeding rates comparable with those achieved when using one-day old-chick stretched skins [25,26,27]. Each feeder unit of the Hemotek system was set to warm the blood meal to 37 °C. A total of 73 feedings were carried out. After each meal, an aliquot of the blood used for feeding was retained and the BTV titer assessed using the Reed and Muench formula [28]. Fed *Culicoides* were immobilized on dry ice, and fully engorged females of *C. imicola* and *C. obsoletus/scoticus* were sorted and counted using a refrigerate chill-table and stereomicroscope. Six to thirty-five blooded midges were assayed after feeding (0 days post-infection, dpi) to establish baseline infection rates and to determine virus load in each midge. The remainder were incubated in cardboard boxes at 25 °C and 80% > HR > 40% for 10 days with ad libitum access to 10% sucrose solution. Those *Culicoides* which survived the incubation period were killed on dry ice and dissected. The heads and thorax/abdomens of each insect were stored separately at −80 °C until further analyses.

#### 2.3.3. Species Identification 

Identification keys, mainly based on wing morphology, and a multiplex PCR, based on internal transcribed spacer 2 ribosomal DNA sequences (ITS2), were used to identify midges [4,29,30,31,32,33]. The morphological identification was performed under the stereomicroscope on a chill table according to Delécolle, Campbell and Pelham-Clinton, and Goffredo and Meiswinkel [29,30,31]. Unlike *C. imicola*, molecular techniques are required to identify *C. obsoletus* and *C. scoticus* at species level [4,32,33]. From each midge head homogenate, total nucleic acids were extracted using the Biosprint^®^ 96 (Qiagen, Hilden, Germany). DNA was used for midge identification while RNA for viral analysis. When species identification was inconclusive, DNA extraction was repeated with the automated Maxwell 16 system (Promega, Madison, WI, USA) with the DNA IQ Casework Sample kit (Promega, Madison, WI, USA) according to manufacturer’s instructions. The ITS-2 segment of ribosomal DNA was amplified using the primers 5.8 SF, 28 SR, Scoticus-194R, MOU-316F, and Montanus-227R [32]. In this study, we refer to “*C. obsoletus/scoticus*” or to “*C. obsoletus* and *C. scoticus*” depending on whether they have been identified by morphological or morphological/molecular tools, respectively. Once identified, midges were individually stored at −80 °C for virus detection. 

#### 2.3.4. Virus Detection

The RT-PCR described by Hofmann et al. [34] was used to detect BTV in midge heads. The VetMAX European BTV Typing Kit (Thermo Fisher Scientific, Waltham, MA, USA) was instead used to determine the BTV serotype. The serogroup-specific RT-PCR [34] used in this study is the test recommended by the Office International des Epizooties (OIE). Its performance in the laboratory has been verified and validated for all known BTV serotypes and most of the strains circulating in the Mediterranean Basin according to the ISO 17025 validation guidelines. To control the entire process, Armored RNA West Nile Virus (HNY1999) (Asuragen, Austin, TX, USA) was included in each PCR reaction as an internal positive control. The threshold cycle value used as a cut off for best discriminating between positive and negative samples was 50. Ct values less than 50 were considered as positive, while those with Ct values equal to 50 as negative. With regard to the Typing kit, according to the manufacturer’s instructions, the Ct value for the best discrimination between positive and negative samples was 40. For each population/strain, the recovery rate (number of BTV positive heads/number of tested heads) was calculated using both the serogroup- and the serotype-specific RT-PCR. The inclusion of the serotype-specific RT-PCR in this trial was necessary since in Sardinia, on the farm where *C. imicola* midges were trapped for BTV-8 competence studies, BTV-4 was circulating at the time of collection. Being a highly specific test, the typing kit has been used to rule out natural infection with BTV-4; therefore, its results were considered when comparing rates involving BTV-8 from the Sardinian population. On the other hand, results obtained with the serogroup-specific RT-PCR, which is more sensitive than the type-specific RT-PCR, were used in most of the recovery rate comparisons made in this study. In analyzing the recovery rate data, more or less conservative approaches were evaluated based on the RT-PCRs cut off levels. The mean Ct values detected immediately after feeding (0 dpi) were also compared with those achieved after the incubation period (10 dpi), and any reduction was interpreted as further evidence of virus replication in the salivary glands [25]. In this study, as in other studies [17], midge heads were used to detect the virus and determine the recovery rates. Though unlikely, there was a chance that salivary glands remained in the thorax when heads were separated. However, since salivary gland barriers have not been described for *Culicoides* species [35], presence of BTV in the head, expressing the complete virus dissemination in the midge, can still be considered a valid indicator of BTV vector competency [17].

### 2.4. Statistical Analysis 

Statistical differences on recovery and feeding rates were investigated through a Bayesian approach, using the Beta distribution. Beta distribution is a type of probability distribution. This distribution represents a family of probabilities and is a versatile way to represent outcomes for percentages or proportions [36]. A 95% confidence interval (C.I.) was calculated for each parameter through the Beta distribution. The comparisons between the recovery and feeding rates were considered significant when the confidence intervals, calculated through the beta distribution, did not overlap.

## 3. Results

### 3.1. Feeding Rate

More than 250,000 *Culicoides* were orally fed in 73 attempts. A total of 6013 midges were engorged after feeding (2203 by Hemotek membrane, 3810 using the cotton method). Of these, 262 were analyzed at 0 dpi and 3218 at 10 dpi. Feeding rates and surviving midges at 10 dpi are reported in Table 1. They were grouped according to collection site, BTV strain, and feeding method. When the Hemotek membrane feeding system was used, not all the *Culicoides* populations investigated were fed successfully. Due to the very low feeding rates obtained, the use of this system was limited to the *Culicoides* populations of Sardinia, Abruzzo, and Calabria and to some BTV strains only (Table 1). The feeding method influenced the *Culicoides* blood-feeding rates, with the cotton pledget (15.88%, 95%CI = 15.43–16.35%) resulting in a significantly higher (*p* < 0.05) blood-feeding rate than the Hemotek Parafilm^®^ membrane (0.97%, 95%CI = 0.93–1.01%). All feeding attempts made using the cotton pledget method were successful, with rates ranging from 9.6% for *C. imicola* (Sardinia BTV-8) to 41.62% for *C. obsoletus/scoticus* of Lazio (BTV-8) (Table 1). With regard to the *Culicoides* species, the *C. imicola* population feeding success rates (1.02%, 95%CI = 0.98–1.07%) were significantly higher (*p* < 0.05) than those of *C. obsoletus/scoticus* (0.07%, 95%CI = 0.04–0.13%) when the Hemotek system was used. Conversely, when cotton pledget was used, a significantly higher rate (*p* < 0.05) of *C. obsoletus/scoticus* (16.78%, 95%CI = 16.11–17.48%) was successfully fed compared to the *C. imicola* (15.06%, 95%CI = 14.45–15.70%).

### 3.2. Competence Studies 

**Figure 2:**
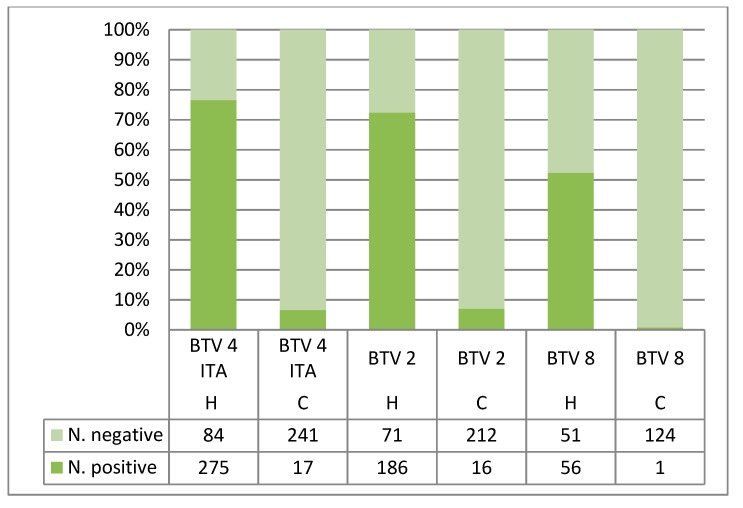
Recovery rate of bluetongue virus strains from *Culicoides imicola* (Sardinia population) fed through different methods: Hemotek device (H), cotton wool pledgets (C).

In this study, the virus titer of the blood meals ranged from 10^5^ TCID_50_/mL (50% Tissue Culture Infective Dose) to 10^6.54^ TCID_50_/mL (Table 2). Of the 262 midges tested immediately after feeding, 225 (85.88%) tested positive for BTV (Table 3). Although in most cases, the differences were not significant, the number of positive midges detected using serogroup-specific RT-PCR were generally higher than those detected by serotype-specific RT-PCR (Table 3 and Table 4). The Sardinian population of *C. imicola* fed via the Hemotek system achieved the best recovery rates regardless of BTV strains. The BTV recovery rate resulting from the serotype-specific RT-PCR at 10 dpi from the Sardinian population of *C. imicola* (66.39%, 95%CI = 62.87–69.74%) fed through the Hemotek membrane was significantly higher than the rate obtained when the same population was fed on cotton wool (3.60%, 95%CI = 2.40–5.39%) (Figure 2; Table 4). The recovery rates remained significantly higher when the more conservative approach, which considered cases where RT-PCR Ct values decreased after the 10–day incubation period, was taken (48.41%, 95%CI = 44.78–52.05% vs. 2.78%, 95% CI = 1.75–4.41%) (Table 5 and Table 6).

With the Hemotek feeding system, the recovery rates of BTV-4 ITA (70.5%; 95%CI = 65.6–75%) and BTV-2 (66.5%, 95%CI = 60.6–72%), obtained at 10 dpi from Sardinian midges were similar (*p* > 0.05). Both rates, however, were higher (*p* < 0.05) than that of BTV-4 MOR (49.2%, 95%CI = 40.4–58%). The recovery rate of BTV-4 ITA was also higher (*p* < 0.05) than that of BTV-8 (52.3%, 95%CI = 42.9–61.6%) (Table 5). If the more conservative approach was used, the recovery rates of BTV-4 ITA, BTV-2, and BTV-8 were similar (*p* > 0.05) but significantly higher (*p* < 0.05) than those of BTV-4 MOR (Table 5 and Table 6).

When midges fed on cotton wool pledgets soaked in infected blood, all *Culicoides* populations tested were susceptible to the BTV strains selected for this trial, with the exception of the *C. obsoletus/scoticus* population of Abruzzo and BTV-4 MOR. However, the BTV recovery rates obtained from *C. imicola* were significantly higher than those from *C. obsoletus/scoticus* (0.83%, 95%CI = 0.41–1.69%; 0.35%, 95%CI = 0.13–1.03%;) when either the serotype-specific RT-PCR (2.55%, 95%CI = 1.81–3.60%) or the 0–10 dpi Ct value reduction (1.64%, 95%CI = 1.07–2.53%) were used as criteria for determining the vector competence. If considered individually, the recovery rate of BTV-2 from the *C. obsoletus/scoticus* population of Lazio (12%; 95%CI = 6.5–21.3%) was significantly (*p* < 0.05) higher than those from the Abruzzo (2.7%; 95%CI = 1.2–5.7%) and Calabria (3.2%; 95%CI = 1.6–6.4%) midge populations. Similarly, the BTV-4 MOR recovery rate from the Abruzzo *Culicoides obsoletus/scoticus* population (0%; 95%CI = 0–2.3%) was significantly lower than those from *C. obsoletus/scoticus* of Lazio (6.3%; 95%CI = 2.5–15%) and *Culicoides imicola* of Calabria (6.9%; 95%CI = 3.6–13%) (Table 4 and Table 6). These differences disappeared (*p* > 0.05%) when the 0–10 dpi Ct value reductions were used to determine the vector susceptibility to different BTV-strain infection (Table 5). This more conservative approach, however, revealed a significant higher recovery rate of BTV-4 ITA from the *C. imicola* population of Sardinia compared to those determined for *C. obsoletus/scoticus* of Abruzzo.

With regard to *C. obsoletus/scoticus* populations either from Lazio (66.2%) or Abruzzo (77.2%), the number of *C. scoticus* collected and identified was higher than that of *C. obsoletus*. With the exception of BTV-2 from the Abruzzo population of *C. obsoletus* and BTV-8 from the Lazio population of *C. scoticus*, all BTV strains examined in this study were recovered from both species with similar rates (*p* > 0.05) (Table 7).

## 4. Discussion

Vector competence is the susceptibility of the vector to infection with the pathogen and the ability of the infected vector to transmit the pathogen to a host during blood feeding. Vector competence is a key component in the vector–pathogen cycle. This study describes the vector competence of different field-collected populations of *C. imicola* and *C. obsoletus* and *C. scoticus* from different sites of Italy, after oral exposure to four bluetongue virus strains (BTV-2, BTV-4 ITA, BTV-4 MOR, and BTV-8). These strains were selected on the basis of their spread and adaptation to different episystems in Europe and the Mediterranean basin. Artificial feeding systems are valuable tools that can be used to investigate vector competence of blood feeding arthropods and have been previously utilized to investigate the vector of *Culicoides* midges and various BTV strains [19,37].

Midges were orally infected using the Hemotek blood feeding system through Parafilm^®^ membrane and the cotton wool pledgets. The results obtained in this study demonstrated that Parafilm^®^ membrane is suitable for *C. imicola* even though, because of its low feeding rate (1–2.03%), a great number of midges should be used for feeding (Table 1). The same cannot be said for species of the Obsoletus complex, since even a great number of midges was not enough to achieve low feeding rates (Table 1). The reluctance of this complex to blood-feed through membrane-based systems has been previously documented [14,15,16]. Because of the low feeding rates achieved using the Hemotek system, vector competence comparisons were mostly made on the data obtained when midges were fed on cotton pledgets. The Hemotek membrane data were used for comparing the susceptibility of the Sardinia population of *C. imicola* to the various BTV strains only. The high feeding rate via the cotton method for both *C. imicola* and *C. obsoletus/scoticus* is in line with the results of other research groups [18,19]. Higher feeding rates, however, do not mean recovery rates. Although acquiring significantly lower feeding rates, the BTV recovery rates from the Sardinian population of *Culicoides imicola* fed through the Hemotek membrane were significantly higher than those fed on cotton pledgets. Venter [19] also found BTV recovery rates from *C. imicola* and *C. bolitinos* fed on cotton pads significantly lower than those fed through the membrane system. The authors hypothesized that the lower recovery rate was probably a consequence of the reduced blood intake (by about 30%) of the midges fed on cotton pads. In their study, the blood meal size was calculated for each method by weighting fed (partially/full engorged not specified) and unfed midges. In this study, only the full engorged females were taken and, thus, the lower dissemination rates cannot be ascribed to a reduced blood intake but to other factors associated to the cotton wool fibers, in particular to their possible interaction with the virus, making it less accessible for midges during feeding.

Another important variable that has been shown to influence the recovery rate following artificial feeding is the virus titer of the blood meal. Paweska [38] stated that there is a strong linear correlation between the virus recovery rates from midges tested immediately after feeding and the virus titer of the blood meal. In this trial, high blood meal titers were used (ranging between 10^5^ TCID_50_/mL and 10^6.54^ TCID_50_/mL) which are similar to the realistic viremic host titers (10^4^–10^6^ TCID_50_/mL) [39,40]. Nonetheless, as revealed by other studies with similar virus titers of blood meals and corroborated in the current study, few midges were still negative when tested immediately after feeding [22,24].

The vector competence of the *Culicoides* populations involved in this trial was determined by testing the heads of midges using serogroup-specific and serotype-specific RT-PCRs after an incubation period of ten days. Positive heads implicated a potential competence for BTV transmission due to the absence of a salivary gland barrier to BTV in *Culicoides* [17,35]. As expected, the serogroup-specific RT-PCR was more sensitive than the type-specific RT-PCR. However, in Sardinia, in the farm where field *C. imicola* midges were trapped for BTV-8 competence studies, BTV-4 was circulating at the time of collection. Thus, to avoid false positive results which could be derived from the use of serogroup-specific RT-PCR in testing BTV-4 positive midges, the type-specific RT-PCR was included in this study. Its results were used in all vector competence comparisons, which included BTV-8 and the Sardinian population of *C. imicola*. For the remainder of the recovery rate comparisons, the results of the serotype-specific RT-PCR assay were used. 

Another important element to take into consideration for the evaluation of the vector competency is the RT-PCR Ct value at 0 and 10 dpi. Ct values at 10 dpi lower than those found at 0 dpi may imply virus replication in the salivary glands [25] and, ultimately, a role for oral transmission. In analyzing the recovery rate data, more or less conservative approaches have been evaluated based on the RT-PCR cut off levels. Because of the great variations of the RT-PCR results and the Ct values observed, to have solid competence data and avoid possible overestimation, a more conservative approach was taken, and only those cases where the RT-PCR Ct values at 10 dpi were lower than the Ct mean values observed at 0 dpi were included in the analysis. Apart from the Abruzzo population of *C. obsoletus/C. scoticus*, which was refractory to BTV-4 MOR infection, all orally infected vector populations tested in this study were able to sustain the BTV strain replication, even that of BTV-4 MOR, a strain which has never been reported in Italy. Unfortunately, the rather low proportion of infected midges obtained after the artificial feeding precluded some competence evaluations and reduced the statistical power of the comparisons.

Overall, BTV recovery rates from *C. imicola* were significantly higher than those recovered from species of the Obsoletus complex. In other words, this study indicated a probably higher susceptibility of *C. imicola* to the selected BTV strains in comparison to the Obsoletus complex populations. To the best of our knowledge, this is the first time that it was possible to compare the competences of either *Culicoides* species. Considering the Obsoletus complex populations, *C. obsoletus* and *C. scoticus* were present with a predominance of *C. scoticus* in either populations. Both species were susceptible to all BTV without any significant difference of recovery rates. Differences of recovery rate among *Culicoides* populations could definitely influence the distribution of BTV strains [5]. Regardless, it must be taken into account that a low susceptibility does not necessarily imply a low vector capacity. Vector competence is just a component of vector capacity, so other elements (high abundance, wide distribution, high biting rate, etc.) are important for virus spread. A low susceptibility could be compensated for by the abundance and wide distribution, as in the case of *C. imicola* and species of the Obsoletus complex in Italy. Susceptibility to infection with BTV can vary in *Culicoides* at species and populations levels [5,37]. It is difficult to understand the cause of the variation in recovery rates between different populations, especially of the same species, since interactions between virus and midge are very complex and poorly investigated. Vector competence could be influenced by hereditary and environmental factors, including the composition of gut microbiota [41]. It has been observed that a blood meal on its own alters the composition/abundance of gut microbiota and influences the expression of antimicrobial peptides [42]. Further studies about transcriptomic analysis and virome and microbiome composition of the Italian populations of *Culicoides* analyzed in this work might allow better understanding these complex and dynamic mechanisms of *Culicoides* infection and vector competence.

Significant differences were also observed between the BTV recovery rates from the *Culicoides* populations tested in this trial. Particularly interesting are the results obtained in the Sardinian population of *C. imicola* after feeding through the Hemotek membrane system. Sardinia has represented and represents the main entrance for most of the Italian BTV incursions. From the Sardinian midge population, the recovery rates of BTV-2, BTV-4 ITAm and BTV-8 were higher than the rate observed for BTV-4 MOR. The higher competence shown by the Sardinian population of *C. imicola* for these 3 BTV strains could explain their successful spread in the island during the BTV-2, BTV-8, and BTV-4 incursions occurring in 2000–2001, 2009, and 2016–2017, respectively [10]. In the same way, it could also be one of the reasons for the possible unsuccessful incursion attempts of BTV-4 MOR. Feeding *Culicoides* midges on cotton wool also evidenced differences in the BTV-4ITA recovery rates from the Sardinian and Abruzzo *Culicoides* populations. The recovery rate from the Sardinia *C. imicola* population (5%, 95%CI 2.99–8.43%) was significantly higher than that obtained from the *C. obsoletus/scoticus* of Abruzzo (0.53%, 95%CI 0.13–2.88%%). This different susceptibility might have contributed to the different impact that the 2016–2017 BTV-4ITA incursion had on the livestock of the two regions. More than 70,000 animals were affected in Sardinia, with an infectious rate of 13.62% (95%CI: 13.53–13.72%), while the affected animals in Abruzzo were only 31, with an infectious rate of 2.84% (95%CI: 2.01–4.01%).

Even though numerous studies on vector competence have been performed, results are in most cases not comparable because of the variety of the infection protocols used. *Culicoides imicola* has been proven to be susceptible to several BTV serotypes, including BTV-2 [43], BTV-4 [20,43], and BTV-8 [22,23]. Species of the Obsoletus complex, although less investigated, have been found to be susceptible to serotypes 1, 4, 8, and 9 [17,18]. The authors of [43] achieved low recovery rates when *C. imicola* midges were fed with BTV-2 (0.5%), BTV-4 (0.2%), and BTV-8 (0%) infected blood through membrane. Relatively low recovery rates of either BTV-8 (<1% for both European and South African strains) or BTV-4 (1.9%), using the virus isolation technique, were also achieved from *C. imicola* fed on 1-day-old chicken membrane [20,22,23]. There were also cases where some BTV strains (BTV-4 and BTV-2) were not even recovered from midges [22]. Paslaru [17] demonstrated the competence of the Obsoletus complex for BTV-8 and BTV-4, obtaining a dissemination rate of 2% for both serotypes. Virus detection was performed by RT-PCR on heads as in our study, but the feeding method and the length and temperature of incubation period were not the same. Thus, also in this case, the variability of important parameters makes it difficult to compare the results. 

## 5. Conclusions

This study has provided some important data on the vector competence of different *C. imicola* and *C. obsoletus/scoticus* populations in Italy in relation to four BTV strains. The results obtained prove that, except for the Abruzzo population of *C. obsoletus/C. scoticus*, which was refractory to BTV-4 MOR infection, all the investigated populations were susceptible to the BTV strains used in the trial, showing, in some instances, significant differences in recovery rates depending on midge population and/or BTV strain. Considering the wide distribution of *C. obsoletus* and *C. scoticus* in Europe, these findings provided evidence that strains like BTV-2 and BTV-4 MOR could have spread all over Europe. Their dissemination failure might have been a consequence of successful vaccination programs and the correct application of restriction measures. Their success was a consequence of contemporaneous appliance of both vaccination of susceptible animals and restricting movement of viremic animals between BT-affected and BT-free zones. The BTV-2 and BTV-4 vaccination campaigns used either modified live vaccines (BTV-2 and BTV-4 outbreaks in Balearic Islands and Corsica in 2000 and 2001, Italy 2003–2004) or inactivated vaccines (BTV-4 outbreaks in Southwest Spain in 2004 and 2005). Both campaigns targeted all susceptible ruminant species, achieved a high degree of herd immunity, and included extensive areas surrounding any active BT outbreaks [44].

## Figures and Tables

**Table 1:** Feeding rate and number of surviving midges at 10 days post-infection (dpi) according to strain, feeding method, and population.

**BTV-4 ITA**		**Fed via Hemotek**				**Fed via Cotton Pledget**			
**SPECIES**	**SITE**	**ENGORGED**	**TOTAL NUMBER**	**FEEDING RATE (%)**	**10 dpi**	**ENGORGED**	**TOTAL NUMBER**	**FEEDING RATE (%)**	**10 dpi**
*Culicoides obsoletus/scoticus*	Abruzzo	5	9139	0.05	**4**	566	3923	14.43	**190**
*Culicoides obsoletus/scoticus*	Lazio					229	752	30.45	**186**
*Culicoides imicola*	Sardinia	1107	54,471	2.03	**359**	330	2078	15.88	**258**
*Culicoides imicola*	Calabria	0	2586	0	**0**	400	2196	18.21	**269**
**BTV-2**		**Fed via Hemotek**				**Fed via Cotton Pledget**			
**SPECIES**	**SITE**	**ENGORGED**	**TOTAL NUMBER**	**FEEDING RATE (%)**	**10 dpi**	**ENGORGED**	**TOTAL NUMBER**	**FEEDING RATE (%)**	**10 dpi**
*Culicoides obsoletus/scoticus*	Abruzzo	4	4131	0.1	**1**	447	4589	9.74	**226**
*Culicoides obsoletus/scoticus*	Lazio					173	516	33.53	**75**
*Culicoides imicola*	Sardinia	719	71,843	1	**257**	351	3376	10.40	**228**
*Culicoides imicola*	Calabria	0	1213	0	**0**	337	2426	13.89	**219**
**BTV-8**		**Fed via Hemotek**				**Fed via Cotton Pledget**			
**SPECIES**	**SITE**	**ENGORGED**	**TOTAL NUMBER**	**FEEDING RATE (%)**	**10 dpi**	**ENGORGED**	**TOTAL NUMBER**	**FEEDING RATE (%)**	**10 dpi**
*Culicoides obsoletus/scoticus*	Abruzzo					174	484	35.95	**110**
*Culicoides obsoletus/scoticus*	Lazio					77	185	41.62	**61**
*Culicoides imicola*	Sardinia	190	57,318	0.33	**107**	153	1593	9.6	**125**
*Culicoides imicola*	Calabria					165	402	41.04	**117**
**BTV-4 MOR**		**Fed via Hemotek**				**Fed via Cotton Pledget**			
**SPECIES**	**SITE**	**ENGORGED**	**TOTAL NUMBER**	**FEEDING RATE (%)**	**10 dpi**	**ENGORGED**	**TOTAL NUMBER**	**FEEDING RATE (%)**	**10 dpi**
*Culicoides obsoletus/scoticus*	Abruzzo					168	720	23.33	**126**
*Culicoides obsoletus/scoticus*	Lazio					87	278	31.29	**64**
*Culicoides imicola*	Sardinia	178	26,863	0.66	**120**				
*Culicoides imicola*	Calabria					153	471	32.48	**116**
*Culicoides obsoletus/scoticus*	**SUBTOTAL**	**9**	**13,270**	**0.07**	**5**	**1921**	**11,447**	**16.78**	**1038**
*Culicoides imicola*	**SUBTOTAL**	**2194**	**214,294**	**1.02**	**843**	**1889**	**12,542**	**15.06**	**1332**
**TOTAL**		**2203**	**227,564**	**0.97**	**848**	**3810**	**23,989**	**15.88**	**2370**

**Table 2:** Titers of bluetongue virus (BTV) strains in blood meals.

Virus	Minimum–Maximum Virus Titer TCID_50_ mL (Number of Blood Meals)
BTV-4 ITA	10^5.3^–10^6.54^ (20)
BTV-2	10^5.01^–10^6.14^ (22)
BTV-8	10^5^–10^6.3^ (16)
BTV-4 MOR	10^5.68^–10^6.39^ (15)

**Table 3:** Virus detection in *Culicoides* tested immediately after feeding on BTV-infected blood at 0 days post-infection.

				Serogroup-Specific RT PCR (Positive Midges Ct < 50)			Serotype-Specific RT PCR (Positive Midges Ct < 40)		
Feeding Method	BTV Strain	Population	Number of Tested	Number of Positive	Recovery Rate (%)	Ct Mean (Min–Max)	Number of Positive	Recovery Rate (%)	Ct Mean (Min–Max)
cotton	4 ITA	*C. obsoletus/scoticus* Abruzzo	16	14	**87.5**	36 (31–44)	/	**/**	/
cotton	4 ITA	*C. obsoletus/scoticus* Lazio	11	11	**100**	38 (36–39)	11	**100**	33 (32–35)
Hemotek	4 ITA	*C. imicola* Sardinia	35	33	**94.3**	37 (35–42)	33	**94.3**	33 (30–36)
cotton	4 ITA	*C. imicola* Sardinia	10	8	**80**	40 (38–42)	8	**80**	35 (33–37)
cotton	4 ITA	*C. imicola* Calabria	15	15	**100**	39 (36–42)	15	**100**	33 (31–36)
cotton	2	*C. obsoletus/scoticus* Abruzzo	15	12	**80**	37 (33–42)	1	**6.7**	39 (39–39)
cotton	2	*C. obsoletus/scoticus* Lazio	11	11	**100**	38 (36–39)	11	**100**	34 (33–35)
Hemotek	2	*C. imicola* Sardinia	21	19	**90.5**	39 (36–43)	18	**85.7**	34 (32–39)
cotton	2	*C. imicola* Sardinia	14	7	**50**	43 (41–44)	4	**28.6**	38 (36–39)
cotton	2	*C. imicola* Calabria	12	12	**100**	40 (35–43)	12	**100**	35 (32–38)
cotton	8	*C. obsoletus/scoticus* Abruzzo	17	12	**70.6**	42 (39–47)	7	**41.2**	35 (34–36)
cotton	8	*C. obsoletus/scoticus* Lazio	7	4	**57.1**	41 (39–42)	2	**28.6**	36 (34–37)
Hemotek	8	*C. imicola* Sardinia	9	/	**/**	/	8	**88.9**	35 (32–37)
cotton	8	*C. imicola* Sardinia	13	/	**/**	/	9	**69.2**	36 (35–37)
cotton	8	*C. imicola* Calabria	15	13	**86.7**	39 (37–41)	13	**86.7**	35 (33–37)
cotton	4 MOR	*C. obsoletus/scoticus* Abruzzo	13	10	**76.9**	42 (38–49)	10	**76.9**	35 (33–38)
cotton	4 MOR	*C. obsoletus/scoticus* Lazio	8	7	**87.5**	40 (37–47)	7	**87.5**	33 (31–35)
Hemotek	4 MOR	*C. imicola* Sardinia	6	6	**100**	36 (34–38)	6	**100**	31 (29–32)
cotton	4 MOR	*C. imicola* Calabria	14	14	**100**	39 (36–41)	13	**92.9**	34 (31–37)

**Table 4:** Virus detection in *Culicoides* (heads) fed on BTV-infected blood at 10 days post-infection.

				Serogroup-Specific RT PCR (Positive Midges Ct < 50)		Serotype-Specific RT PCR (Positive Midges Ct < 40)	
Feeding Method	BTV Strain	Population	Number of Tested	Number of Positive	Ct Mean (Min–Max)	Number of Positive	Ct Mean (Min–Max)
Hemotek	4 ITA	*C. obsoletus*/*scoticus* Abruzzo	4	0	/	/	/
cotton	4 ITA	*C. obsoletus*/*scoticus* Abruzzo	190	9	40 (34–46)	1	29 (29–29)
cotton	4 ITA	*C. obsoletus*/*scoticus* Lazio	186	18	41 (25–47)	2	33 (27–39)
Hemotek	4 ITA	*C. imicola* Sardinia	359	275	32 (24–48)	253	30 (23–39)
cotton	4 ITA	*C. imicola* Sardinia	258	17	36 (27–41)	9	33 (27–38)
cotton	4 ITA	*C. imicola* Calabria	269	15	40 (26–47)	6	33 (24–37)
Hemotek	2	*C. obsoletus*/*scoticus* Abruzzo	1	0	/	/	/
cotton	2	*C. obsoletus*/*scoticus* Abruzzo	226	6	38 (27–42)	1	30 (30–30)
cotton	2	*C. obsoletus*/*scoticus* Lazio	75	9	38 (33–40)	2	36 (33–38)
Hemotek	2	*C. imicola* Sardinia	257	186	32 (22–47)	171	29 (23–39)
cotton	2	*C. imicola* Sardinia	228	16	40 (32–45)	12	35 (30–39)
cotton	2	*C. imicola* Calabria	219	7	41 (28–45)	2	32 (25–38)
cotton	8	*C. obsoletus*/*scoticus* Abruzzo	110	4	43 (39–46)	1	38 (38–38)
cotton	8	*C. obsoletus*/*scoticus* Lazio	61	2	42 (39–44)	0	/
Hemotek	8	*C. imicola* Sardinia	107	/	/	56	30 (27–38)
cotton	8	*C. imicola* Sardinia	125	/	/	1	30 (30–30)
cotton	8	*C. imicola* Calabria	117	12	40 (31–44)	1	34 (34–34)
cotton	4 MOR	*C. obsoletus*/*scoticus* Abruzzo	126	0	/	/	/
cotton	4 MOR	*C. obsoletus*/*scoticus* Lazio	64	4	40 (38–43)	0	/
Hemotek	4 MOR	*C. imicola* Sardinia	120	62	38 (31–45)	59	32 (27–38)
cotton	4 MOR	*C. imicola* Calabria	116	8	38 (33–43)	6	32 (29–39)

**Table 5:** Recovery rates of bluetongue virus strains from different Italian vector populations orally infected according to different RT-PCR cut-off levels.

				Serogroup-Specific RT-PCR		Serotype-Specific RT-PCR	
Feeding Method	BTV Strain	Population	Number of Tested	Number of Positive with Ct < 50 (Recovery Rate%)	Number of Positive with Ct < Mean Ct at 0 dpi (Recovery Rate%)	Number of Positive with Ct < 40 (Recovery Rate%)	Number of Positive with Ct < Mean Ct at 0 dpi (Recovery Rate%)
cotton	4 ITA	*C. obsoletus/scoticus* Abruzzo	190	9 (4.7)	1 (0.5)	1 (0.5)	/
cotton	4 ITA	*C. obsoletus/scoticus* Lazio	186	18 (9.7)	2 (1.1)	2 (1.1)	1 (0.5)
Hemotek	4 ITA	*C. imicola* Sardinia	359	275 (76.6)	201 (56)	253 (70.5)	165 (46)
cotton	4 ITA	*C. imicola* Sardinia	258	17 (6.6)	13 (5)	9 (3.5)	6 (2.3)
cotton	4 ITA	*C. imicola* Calabria	269	15 (5.6)	4 (1.5)	6 (2.2)	1 (0.4)
cotton	2	*C. obsoletus/scoticus* Abruzzo	226	6 (2.7)	2 (0.9)	1 (0.4)	1 (0.4)
cotton	2	*C. obsoletus/scoticus* Lazio	75	9 (12.0)	2 (2.7)	2 (2.7)	1 (1.3)
Hemotek	2	*C. imicola* Sardinia	257	186 (72.4)	148 (57.6)	171 (66.5)	131 (51.4)
cotton	2	*C. imicola* Sardinia	228	16 (7.0)	11 (4.8)	12 (5.3)	10 (4.4)
cotton	2	*C. imicola* Calabria	219	7 (3.2)	2 (0.9)	2 (0.9)	1 (0.5)
cotton	8	*C. obsoletus/scoticus* Abruzzo	110	4 (3.6)	2 (1.8)	1 (0.9)	0 (0)
cotton	8	*C. obsoletus/scoticus* Lazio	61	2 (3.3)	1 (1.6)	0 (0)	0 (0)
Hemotek	8	*C. imicola* Sardinia	107	/	/	56 (52.3)	54 (50.5)
cotton	8	*C. imicola* Sardinia	125	/	/	1 (0.8)	1 (0.8)
cotton	8	*C. imicola* Calabria	117	12 (10.3)	2 (1.7)	1 (0.9)	1 (0.9)
cotton	4 MOR	*C. obsoletus/scoticus* Abruzzo	126	0 (0)	0 (0)	0 (0)	0 (0)
cotton	4 MOR	*C. obsoletus/scoticus* Lazio	64	4 (6.3)	2 (3.1)	0 (0)	0 (0)
Hemotek	4 MOR	*C. imicola* Sardinia	120	62 (51.7)	16 (13.3)	59 (49.2)	21 (17.5)
cotton	4 MOR	*C. imicola* Calabria	116	8 (6.9)	5 (4.3)	6 (5.2)	4 (3.4)

**Table 6:** Comparison of the recovery rates (serogroup-specific/serotype-specific RT-PCR positive heads/tested heads at 10 days post-infection) from different Italian vector populations, orally infected with BTV strains.

			Recovery Rate % (Confidence Intervals %)			
RT-PCR	Population	Feeding Method	BTV-4 ITA	BTV-2	BTV-8	BTV-4 MOR
Serogroup-specific RT-PCR	*C. obsoletus/scoticus* Abruzzo	Cotton	0.5 (0.1–2.9)	0.9 (0.3–3.1)	1.8 (0.6–6.4)	0 (0–2.3)
	*C. obsoletus/scoticus* Lazio	Cotton	1.1 (0.3–3.8)	2.7 (0.8–9.2)	1.6 (0.4–8.7)	3.1 (1–10.7)
	*C. imicola* Sardinia	Hemotek	56 (50.8–61)	57.6 (51.5–63.5)	/	13.3 (8.4–20.6)
		Cotton	5 (3–8.4)	4.8 (2.7–8.4)	/	/
	*C. imicola* Calabria	Cotton	1.5 (0.6–3.7)	0.9 (0.3–3.2)	1.7 (0.5–6)	4.3 (1.9–9.7)
Serotype-specific RT-PCR	*C. obsoletus/scoticus* Abruzzo	Cotton	/	0.4 (0.1–2.4)	0 (0–2.7)	0 (0–2.3)
	*C. obsoletus/scoticus* Lazio	Cotton	0.5 (0.1–2.9)	1.3 (0.3–7.1)	0 (0–4.7)	0 (0–4.5)
	*C. imicola* Sardinia	Hemotek	46 (40.9–51.1)	51.4 (44.9–57)	50.5 (41.1–59.8)	17.5 (11.8–25.3)
		Cotton	2.3 (1.1–5)	4.4 (2.4–7.9)	0.8 (0.2–4.3)	/
	*C. imicola* Calabria	Cotton	0.4 (0.1–2)	0.5 (0.1–2.5)	0.9 (0.2–4.6)	3.4 (1.4–8.5)

**Table 7:** Recovery rate (serogroup-specific RT-PCR) and relative 95% confidence interval of *Culicoides obsoletus* and *C. scoticus* fed on BTV-infected blood at 0 and 10 days post-infection.

Dpi	BTV Strain	Site	Species	Number of Positive/Number of Tested	Recovery Rate %	Confidence Intervals %	CT Mean (Min–Max)
**0**	4 ITA	Abruzzo	*C. obsoletus*	5/6	83.3	/	37 (32–44)
			*C. scoticus*	8/9	88.9	/	34 (31–40)
			*C. obsoletus/scoticus* *	1/1	100	/	41 (41–41)
		Lazio	*C. obsoletus*	1/1	100	/	37 (37–37)
			*C. scoticus*	10/10	100	/	38 (36–39)
	2	Abruzzo	*C. obsoletus*	2/2	100	/	38 (33–42)
			*C. scoticus*	10/12	83.3	/	37 (33–42)
		Lazio	*C. obsoletus*	2/2	100	/	38 (36–39)
			*C. scoticus*	9/9	100	/	38 (36–39)
	8	Abruzzo	*C. obsoletus*	6/8	75	/	42 (39–45)
			*C. scoticus*	4/7	57.1	/	42 (39–47)
			*C. obsoletus/scoticus* *	2/2	100	/	41 (40–42)
		Lazio	*C. obsoletus*	6/6	100	/	41 (41–42)
			*C. scoticus*	1/1	100	/	39 (39–39)
	4 MOR	Abruzzo	*C. obsoletus*	3/5	60	/	43 (40–48)
			*C. scoticus*	7/8	87.5	/	42 (38–49)
		Lazio	*C. obsoletus*	3/4	75	/	39 (38–41)
			*C. scoticus*	4/4	100	/	40 (37–47)
**10**	4 ITA	Abruzzo	*C. obsoletus*	1/14	7.1	1.7–31.9	34 (34–34)
			*C. scoticus*	8/173	4.6	2.4–8.9	41 (37–46)
			*C. obsoletus/scoticus* *	0/3	0	/	/
		Lazio	*C. obsoletus*	3/39	7.7	2.8–20.4	41 (38–43)
			*C. scoticus*	15/146	10.3	6.4–16.3	40 (25–47)
			*C. obsoletus/scoticus* *	0/1	0	/	/
	2	Abruzzo	*C. obsoletus*	0/46	0	0.1–7.5	/
			*C. scoticus*	6/178	3.4	1.6–7.2	38 (27–42)
			*C. obsoletus/scoticus* *	0/2	0	/	/
		Lazio	*C. obsoletus*	2/16	12.5	3.8–36.4	36 (33–39)
			*C. scoticus*	7/59	11.9	5.9–22.6	39 (37–40)
	8	Abruzzo	*C. obsoletus*	1/45	2.2	0.5–11.5	44 (44–44)
			*C. scoticus*	3/63	4.8	1.7–13.1	42 (39–46)
			*C. obsoletus/scoticus* *	0/2	0	/	/
		Lazio	*C. obsoletus*	2/46	4.3	1.3–14.5	42 (39–44)
			*C. scoticus*	0/15	0	0.2–20.6	/
	4 MOR	Abruzzo	*C. obsoletus*	0/22	0	0.1–14.8	/
			*C. scoticus*	0/103	0	0–3.5	/
			*C. obsoletus/scoticus* *	0/1	0	/	/
		Lazio	*C. obsoletus*	2/28	7.1	2.2–22.8	41 (38–43)
			*C. scoticus*	2/36	5.6	0.1–9.5	40 (39–41)

* species identification inconclusive.
